# Supplementary material for: Serum peptidomic profiling and peptide mass fingerprinting reveal signatures associated with peroxisomal and mitochondrial pathways in MMVD-associated cardiorenal syndrome in dogs
Source: PLoS One. 2026 May 15;21(5):e0348233. doi: 10.1371/journal.pone.0348233 (PMC13178898; doi:10.1371/journal.pone.0348233)
Supplement: S1 Table — Abbreviations: Healthy, healthy control; MMVD B1, myxomatous mitral valve disease stage B1; MMVD C WOAZ, MMVD stage C without azotemia; MMVD C WAZ, MMVD stage C with azotemia; CKD stage 2, chronic kidney disease at IRIS stage 2. Data are presented as proportions (percentages). (DOCX) [file pone.0348233.s001.docx]

| Medication | Healthy  (n = 15) | MMVD B1  (n = 10) | MMVD C WOAZ  (n = 15) | MMVD C WAZ  (n = 13) | CKD stage 2  (n = 11) |
| --- | --- | --- | --- | --- | --- |
| Pimobendan | 0 | 0 | 15 (100%) | 13 (100%) | 0 |
| ACEIs | 0 | 0 | Ramipril 6 (40%)  Benazepril 3 (20%) | Ramipril 2 (15%) | 0 |
| Furosemide | 0 | 0 | 15 (100%) | 13 (100%) | 0 |
| Spironolactone | 0 | 0 | 9 (60%) | 7 (54%) | 0 |
| Sildenafil | 0 | 0 | 3 (20%) | 5 (38%) | 0 |

Supplementary table 1. Medication of the enrolled dog

Abbreviations: Healthy, healthy control; MMVD B1, MMVD dogs at stage B1; MMVD C WOAZ, MMVD stage C without azotemia; MMVD C WAZ, MMVD stage C complicated by azotemia; CKD stage 2, CKD at IRIS stage 2, Data are reported as the proportion (percent).
