# Supplementary material for: Serum peptidomic profiling and peptide mass fingerprinting reveal signatures associated with peroxisomal and mitochondrial pathways in MMVD-associated cardiorenal syndrome in dogs
Source: PLoS One. 2026 May 15;21(5):e0348233. doi: 10.1371/journal.pone.0348233 (PMC13178898; doi:10.1371/journal.pone.0348233)
Supplement: S2 Table — Abbreviations: RBC, red blood cell; Hb, hemoglobin; Hct, hematocrit; MCV, mean corpuscular volume; MCH, mean corpuscular hemoglobin; MCHC, mean corpuscular hemoglobin concentration; WBC, white blood cell; ALT, alanine aminotransferase; ALP, alkaline phosphatase; BUN, blood urea nitrogen; Healthy, healthy control; MMVD B1, myxomatous mitral valve disease stage B1; MMVD C WOAZ, MMVD stage C without azotemia; MMVD C WAZ, MMVD stage C with azotemia; CKD stage 2, chronic kidney disease at IRIS stage 2. Statistical differences among groups were assessed using the Kruskal–Wallis test followed by the Mann–Whitney U test. Superscript letters indicate statistically significant differences between groups (P < 0.05). Data are presented as medians with interquartile ranges. Reference intervals were obtained from the Clinical Laboratory of Prasu Arthorn Veterinary Teaching Hospital. (DOCX) [file pone.0348233.s002.docx]

Supplementary table 2. Complete blood count and blood chemistry profiles of dogs in the experimental groups

| Parameters | Healthy  (n = 15) | MMVD B1  (n = 10) | MMVD C WOAZ  (n = 15) | MMVD C WAZ  (n = 13) | CKD stage 2  (n = 11) | Reference intervals | P-value |
| --- | --- | --- | --- | --- | --- | --- | --- |
| RBC (x10^6^ cell/μL) | 7 [5.3 -6.8]^a^ | 6 [4.9 - 6.6]^b^ | 7 [6 - 7.6]^abc^ | 6 [5.4 - 7.0] | 6 [4.1 - 6.6]^c^ | 5-9 | 0.043 |
| Hb (g/dL) | 16 [13.5 - 16.2] | 14 [12.4 - 15.5]^a^ | 16 [14.8 - 17.7]^abc^ | 15 [13.4 - 16.1]^b^ | 13 [12.4 - 15.2]^c^ | 10-18 | 0.016 |
| Hct (%) | 47 [40 - 49.8] | 43 [37 - 45]^a^ | 48 [42.7 - 54.1]^ab^ | 42 [39.7 - 47.5] | 40 [32.7 - 45.7]^b^ | 35-55 | 0.039 |
| MCV (fL) | 71 [70 - 74] | 72 [68 -76] | 70 [68 - 73] | 70 [65 - 71] | 71 [68 - 72] | 60-77 | 0.411 |
| MCH (pg) | 24 [23.4 - 24.6] | 24 [22.8 - 25] | 24 [23.4 - 24.5] | 24 [22.2 - 24.5] | 24 [23 - 24.5] | 20-25 | 0.702 |
| MCHC (g/dL) | 34 [32.4 - 33.8] | 33 [32.7 - 34] | 34 [33.3 - 34] | 34 [33.3 - 34] | 34 [33.1 - 34] | 32-36 | 0.440 |
| Platelet  (x10^3^cell/μL) | 296 [217 - 364] | 307 [223 - 425] | 269 [169 - 297] | 341 [210 - 466] | 317 [233 - 424] | 200-500 | 0.375 |
| WBC  (x10^3^cell/uL) | 9.8 [6.5 - 17.2] | 9.4 [7.4 - 12.3] | 10 [7.7 - 13.8] | 8.0 [6.5 - 18.8] | 10.2 [8.7 - 12.4] | 6-17 | 0.998 |
| Neutrophils (x10^3^cell/uL) | 7.1 [4.6 - 12.7] | 6.7 [5.3 - 8.3] | 7.7 [6.0 - 9.8] | 5.7 [4.7 - 13.9] | 7.4 [6.0 - 9.4] | 3.0-11.5 | 0.987 |
| Eosinophils (x10^3^cell/uL) | 0.2 [0.16 - 0.43] | 0.34 [0.16 - 0.54] | 0.3 [0.08 - 0.43] | 0.17 [0.09 - 0.39] | 0.26 [0.08 - 0.51] | 1.0-4.8 | 0.871 |
| Basophils (x10^3^cell/uL) | 0 | 0 | 0 | 0 | 0 | 0-0.1 | 1.000 |
| Lymphocytes (x10^3^cell/uL) | 1.9 [1.7 -2.1] | 1.9 [1.3 - 2.5] | 1.9 [1.2 - 2.4] | 1.9 [1.4 - 2.9] | 1.7 [1.4 - 2.5] | 1.0-4.8 | 0.990 |
| Monocytes (x10^3^cell/uL) | 0.39 [0.27 - 1.0] | 0.33 [0.22 - 0.47] | 0.53 [0.26 - 0.89] | 0.6 [0.35 - 1.0] | 0.44 [0.25 - 0.58] | 0.15-1.35 | 0.292 |

Abbreviations: RBC, Red blood cell; Hb, Hemoglobin; Hct, Hematocrit; MCV, Mean corpuscular volume. MCH, Mean corpuscular hemoglobin; MCHC, Mean corpuscular hemoglobin concentration; WBC, White blood cell; Healthy, healthy control; MMVD B1, MMVD dogs at stage B1; MMVD C WOAZ, MMVD stage C without azotemia; MMVD C WAZ, MMVD stage C complicated by azotemia; CKD stage 2, CKD at IRIS stage 2. The statistically significant differences within each group were tested using the Kruskal–Walli’s test and Mann–Whitney U test. a Statistically significant difference compared between groups (P < 0.05). b Statistically significant difference compared between groups (P < 0.05). c Statistically significant difference compared between groups (P < 0.05). The results are presented as medians with interquartile ranges. A reference intervals were obtained from the Clinical Laboratory of Prasu Arthorn Veterinary Teaching Hospital Reference Values.

| Parameters | Healthy  (n = 15) | MMVD B1  (n = 10) | MMVD C WOAZ  (n = 15) | MMVD C WAZ  (n = 13) | CKD stage 2  (n = 11) | Reference intervals | P-value |
| --- | --- | --- | --- | --- | --- | --- | --- |
| ALT (IU/L) | 65 [40 -71] | 70 [54 -92] | 69 [43 -96] | 41 [32 - 64] | 56 [35 -114] | 10-100 | 0.286 |
| ALP (IU/L) | 87 [22 - 100] | 95 [44 - 168] | 64 [39 - 150] | 75 [26 - 145]^c^ | 44 [27 - 80] | 23-212 | 0.419 |
| Creatinine (mg/dL) | 1.2 [1.14 - 1.3]^a^ | 1.2 [1.1 - 1.39]^b^ | 1.3 [1 - 1.46]^c^ | 1.9 [1.82 - 2.0]^abc^ | 1.95 [1.89 - 2.0] ^abc^ | 0.50-1.80 | 0.000 |
| BUN (mg/dL) | 35 [33 - 38]^a^ | 34.5 [23 - 37]^b^ | 23 [20 -37]^ac^ | 64 [51 - 100]^abc^ | 50 [22 - 103] | 7-27 | 0.003 |
| Total protein (g/dL) | 7.0 [6.4 - 7.6] | 7.5 [6.9 - 7.6] | 6.8 [6.7 - 7.6] | 6.8 [6.4 - 7.7] | 6.9 [6.2 - 7.4] | 5.2-8.2 | 0.550 |
| Albumin (g/dL) | 2.8 [2.7 -2.9]^a^ | 2.9 [2.7 -3.3] | 3.1 [2.9 - 3.3]^ab^ | 3 [2.7 - 3.15] | 2.6 [2.4 - 3] ^b^ | 2.7 -3.8 | 0.036 |
| Globulin (g/dL) | 4.3 [3.6 - 5] | 4.4 [4.1 - 4.6] | 4.1 [3.5 - 4.6] | 3.8 [3.45 - 5] | 4.2 [3.4 - 4.6] | 2.3–5.2 | 0.606 |
| Glucose g/dL | 87 [76 -87] | 87 [76 -95] | 93 [83 - 97] | 91 [83 - 101] | 91 [88 - 98] | 77-125 | 0.535 |

Supplementary table 2 (continue). Complete blood count and blood chemistry profiles of dogs in the experimental groups

Abbreviations: ALT, Alanine aminotransferase; ALP, Alkaline phosphatase; BUN, Blood urea nitrogen. Healthy, healthy control; MMVD B1, MMVD dogs at stage B1; MMVD C WOAZ, MMVD stage C without azotemia; MMVD C WAZ, MMVD stage C complicated by azotemia; CKD stage 2, CKD at IRIS stage 2. The statistically significant differences within each group were tested using the Kruskal–Walli’s test and Mann–Whitney U test. a Statistically significant difference compared between groups (P < 0.05). b Statistically significant difference compared between groups (P < 0.05). c Statistically significant difference compared between groups (P < 0.05). The results are presented as medians with interquartile ranges. A reference intervals were obtained from the Clinical Laboratory of Prasu Arthorn Veterinary Teaching Hospital Reference Values.
