# Supplementary material for: Serum peptidomic profiling and peptide mass fingerprinting reveal signatures associated with peroxisomal and mitochondrial pathways in MMVD-associated cardiorenal syndrome in dogs
Source: PLoS One. 2026 May 15;21(5):e0348233. doi: 10.1371/journal.pone.0348233 (PMC13178898; doi:10.1371/journal.pone.0348233)
Supplement: S3 Table — Echocardiographic indices were normalized using Cornell’s allometric scaling method. Abbreviations: VHS, vertebral heart score; VLAS, vertebral left atrial score; IVSd, interventricular septal thickness at end-diastole; LVIDd, left ventricular internal diameter at end-diastole; LVPWd, left ventricular posterior wall thickness at end-diastole; IVSs, interventricular septal thickness at end-systole; LVIDs, left ventricular internal diameter at end-systole; LVPWs, left ventricular posterior wall thickness at end-systole; LA, left atrium; AO, aorta; LA:AO, left atrium-to-aorta ratio; FS, fractional shortening; Healthy, healthy control; MMVD B1, myxomatous mitral valve disease stage B1; MMVD C WOAZ, MMVD stage C without azotemia; MMVD C WAZ, MMVD stage C with azotemia; CKD stage 2, chronic kidney disease at IRIS stage 2. Statistical differences among groups were evaluated using the Kruskal–Wallis test, with post hoc pairwise comparisons performed using the Mann–Whitney U test. Superscript letters indicate statistically significant differences between groups for each variable (P < 0.05); groups sharing the same letter are significantly different. Data are presented as medians with interquartile ranges. (DOCX) [file pone.0348233.s003.docx]

Supplementary table 3. Thoracic radiographic findings, echocardiographic indices, and systolic blood pressure of the enrolled dog

| Parameters | Healthy  (n = 15) | MMVD B1  (n = 10) | MMVD C WOAZ  (n = 15) | MMVD C WAZ  (n = 13) | CKD stage 2  (n = 11) | P value |
| --- | --- | --- | --- | --- | --- | --- |
| Thoracic radiography | | | | | | |
| VHS | 10.2 [9.8 - 10.5]^ac^ | 10.7 [10.4 - 10.8]^ab^ | 12 [11.8 - 13]^ab^ | 12 [11.8 - 12.9]^ab^ | 10.2 [9 - 10.4]^b^ | < 0.001 |
| VLAS | 2.2 [1.8 - 2.3]^a^ | 2.5 [2.2 - 2.7]^ab^ | 3.4 [2.8 -3.6]^ab^ | 3.2 [2.8 - 3.2]^ab^ | 2.1 [1.4 -2.3] | < 0.001 |
| Echocardiography | | | | | | |
| IVSd (cm) | 0.7 [0.65 - 0.7] | 0.69 [0.63 - 0.78] | 0.7 [0.5 - 0.8] | 0.7 [0.7 - 0.8] | 0.67 [0.65 - 0.7] | 0.771 |
| LVIDd (cm) | 1.9 [1.7 - 2.4]^a^ | 2.25 [1.7 - 2.4]^b^ | 3.0 [2.8 - 3.3]^ab^ | 3.3 [2.8 - 3.0]^abc^ | 1.7 [1.7 - 2.4]^c^ | < 0.001 |
| LVPWd (cm) | 0.7 [0.65 - 0.7]^ab^ | 0.68 [0.57 - 0.75]^cd^ | 0.6 [0.5 - 0.7]^a^ | 0.5 [0.5 - 0.62]^ace^ | 0.7 [0.67 -0.7]^de^ | 0.011 |
| IVSs (cm) | 0.9 [0.8 - 1.2] | 0.88 [0.85 - 1.2] | 1.2 [0.9 - 1.9] | 1.1 [1.0 - 1.2] | 0.87 [0.8 - 1.2] | 0.054 |
| LVIDs (cm) | 0.9 [0.87- 1.1]^a^ | 0.95 [0.88 - 1.2]^b^ | 1.3 [1.2 - 1.5]^abc^ | 1.3 [1.2 - 1.4]^abd^ | 0.9 [0.84 - 1.1]^cd^ | 0.000 |
| LVPWs (cm) | 0.9 [0.9 - 1.2] | 0.95 [0.9 - 1.1] | 1.1 [1 - 1.2] | 1.1 [0.95 - 1.2] | 0.9 [0.9 - 1.2] | 0.343 |
| NLVIDd (cm/weight (kg)^0.294^) | 1.3 [1.2 - 1.4]^a^ | 1.52 [1.4 - 1.57]^ab^ | 1.88 [1.83 - 2.0]^ab^ | 1.95 [1.88 - 2.15]^ab^ | 1.3 [1.1 - 1.3]^b^ | < 0.001 |
| La (cm) | 1.4 [1.35 - 1.6]^a^ | 1.4 [1.3 - 1.6]^b^ | 2.4 [2.2 - 3.4]^abc^ | 2.5 [2.3 -3.15]^abd^ | 1.4 [1.2 -1.6]^cd^ | < 0.001 |
| Ao (cm) | 1.1 [1.1 - 1.3] | 1.1 [0.87-1.1] | 1.1 [1 - 1.5] | 1.3 [1.1 - 1.45] | 1.1 [1 - 1.6] | 0.172 |
| La/AO | 1.2 [1.1- 1.3]^a^ | 1.41 [1.22 - 1.51]^b^ | 2.2 [1.8 - 2.4]^abc^ | 1.92 [1.76 - 2.19]^abc^ | 1.27 [1 - 1.41]^bc^ | < 0.001 |
| %FS (%) | 50 [40 - 55] | 51 [40 - 55] | 54 [47 - 62] | 54 [48 - 57] | 50 [36 - 63] | 0.439 |
| MV E vel (m/s) | 0.9 [0.78 - 1.1]^a^ | 0.77 [0.66- 0.92]^b^ | 1.2 [1.1 - 1.5]^abc^ | 1.2 [0.87 - 1.5]^b^ | 0.9 [0.78 - 0.9]^c^ | 0.000 |
| MV A vel (m/s) | 0.9 [0.7 - 0.98] | 0.85 [0.77 - 1.01] | 0.93 [0.9 - 1.1] | 0.99 [0.89 - 1.14]^a^ | 0.7 [0.7 - 0.98]^a^ | 0.039 |
| MV E/A | 1.1 [0.2 - 1.2] | 0.93 [0.67 - 1.3] | 1.24 [0.98 - 1.66] | 1.24 [0.77 - 1.5] | 1.16 [0.24-1.29] | 0.407 |
| MR Vmax (m/s) | 0^a^ | 5.65 [5.37 - 5.82]^a^ | 5.64 [5.37 - 5.9]^a^ | 5.7 [5.47 - 6.0]^a^ | 0 | < 0.001 |
| AV Vmax (m/s) | 1.2 [0.94 - 1.21]^a^ | 1.0 [0.94 - 1.21]^b^ | 1.4 [1.1 - 1.58]^abc^ | 1.0 [0.8 - 1.2]^c^ | 0.94 [0.84 - 1.24]^c^ | 0.008 |
| PV Vmax (m/s) | 0.9 [0.85 - 0.9] | 0.89 [0.84 - 0.9] | 1.23 [0.87 - 1.31] | 0.9 [0.8 - 1.22] | 0.88 [0.8 - 0.98] | 0.164 |
| TR Vmax (m/s) | 0^a^ | 0^b^ | 2.87 [0 - 2.9]^abc^ | 2.38 [0 - 3.17]^abc^ | 0^c^ | < 0.001 |
| Blood pressure | | | | | | |
| Systolic blood pressure (mmHg) | 129 [125 - 134] | 130[127 - 133] | 131[130 - 134] | 130 [128 -132] | 131 [128-133] | 0.599 |

The echocardiographic indices were normalized by using Cornell’s allometric scaling method. Abbreviations: VHS, Vertebral heart score; VLAS, Vertebral Left Atrial Score; IVSd, Interventricular septal thickness at end diastole; LVIDd, Left ventricular internal diameter at end diastole; LVPWd, Left ventricular posterior wall thickness at end diastole; IVSs, Interventricular septal thickness at end systole; LVIDs, Left ventricular internal diameter at end systole; LVPWs, Left ventricular posterior wall thickness at end systole; LA, Left atrium; AO, Aorta; LA:AO, Left atrium to aorta ratio; FS, fractional shortening, Healthy, healthy control; MMVD B1, MMVD dogs at stage B1; MMVD C WOAZ, MMVD stage C without azotemia; MMVD C WAZ, MMVD stage C complicated by azotemia; CKD stage 2, CKD at IRIS stage 2. Statistical differences within each variable were analyzed using the Kruskal-Wallis test, with post hoc pairwise comparisons conducted using the Mann-Whitney U test. Significant differences (P < 0.05) between groups for each variable are indicated by same letters (a, b, c, d) within the same row. Groups sharing the same letter are significantly different. The results are presented as medians with interquartile ranges.
