# Supplementary material for: Serum peptidomic profiling and peptide mass fingerprinting reveal signatures associated with peroxisomal and mitochondrial pathways in MMVD-associated cardiorenal syndrome in dogs
Source: PLoS One. 2026 May 15;21(5):e0348233. doi: 10.1371/journal.pone.0348233 (PMC13178898; doi:10.1371/journal.pone.0348233)
Supplement: S4 Table — Abbreviations: Healthy, healthy control; MMVD B1, myxomatous mitral valve disease stage B1; MMVD C WOAZ, MMVD stage C without azotemia; MMVD C WAZ, MMVD stage C with azotemia; CKD stage 2, chronic kidney disease at IRIS stage 2. Statistical differences among groups were evaluated using the Kruskal–Wallis test with post hoc pairwise comparisons performed using the Mann–Whitney U test. Categorical data were analyzed using the chi-square test. Superscript letters indicate statistically significant differences between groups for each variable (P < 0.05); groups sharing the same letter are significantly different. Data are presented as medians with interquartile ranges. (DOCX) [file pone.0348233.s004.docx]

Supplementary table 4. Urinalysis indices of the enrolled dog

| Parameters | Healthy  (n = 15) | MMVD B1  (n = 10) | MMVD C WOAZ  (n = 15) | MMVD C WAZ  (n = 13) | CKD stage 2  (n = 11) | P-value |
| --- | --- | --- | --- | --- | --- | --- |
| Color (no. of sample) | Yellow (8), pale yellow (7) | Yellow (6), pale yellow (4) | Yellow (9), pale yellow (6) | Yellow (8), pale yellow (5) | Yellow (7), pale yellow (4) | 0.28097 |
| Clarity (no. of sample) | Clear (7), Slightly cloudy (8) | Clear (7), Slightly cloudy (3) | Clear (7), Slightly cloudy (8) | Clear (8), Slightly cloudy (5) | Clear (5), Slightly cloudy (6) | 0.89380 |
| USG | 1.041 [1.034 - 1.046] | 1.025 [1.019 - 1.033] | 1.020[1.015 - 1.026]^a^ | 1.014 [1.010 - 1.017]^a^ | 1.015 [1.012 - 1.025]^a^ | <0.001 |
| Urine pH | 6 [5.6 - 6.4] | 6.7 [5.8 -7.4] | 6.5 [6.1 - 6.8] | 6.2 [5.9 - 6.9] | 6.5 [6.2 - 6.9] | 0.062 |
| Protein dipstick (no. of sample) | Negative (8),  trace (7) | Negative (6),  trace (4) | Negative (9),  trace (6) | Negative (10),  trace (3) | Negative (8),  trace (3) | 0.79676 |
| Glucose  (no. of sample) | Negative (15) | Negative (10) | Negative (15) | Negative (13) | Negative (11) | 1.00000 |
| Ketone dip stick (no. of sample) | Negative (15) | Negative (10) | Negative (15) | Negative (13) | Negative (11) | 1.00000 |
| Bilirubin dipstick  (no. of sample) | Negative (7), trace (8) | Negative (7), trace (3) | Negative (9), trace (6) | Negative (8), trace (5) | Negative (8), trace (3) | 0.79676 |
| Hemoglobin dipstick  (no. of sample) | Negative (15) | Negative (10) | Negative (12), trace (3) | Negative (8), trace (5) | Negative (9), trace (2) | 0.38392 |
| Urobilinogen dipstick  (no. of sample) | Normal (15) | Normal (10) | Normal (15) | Normal (13) | Normal (11) | 1.00000 |
| Nitrite dipstick  (no. of sample) | Negative (15) | Negative (10) | Negative (15) | Negative (13) | Negative (11) | 1.00000 |
| Leukocytes dipstick (no. of sample) | Negative (15) | Negative (10) | Negative (15) | Negative (13) | Negative (11) | 1.00000 |

Abbreviations: Healthy, healthy control; MMVD B1, MMVD dogs at stage B1; MMVD C WOAZ, MMVD stage C without azotemia; MMVD C WAZ, MMVD stage C complicated by azotemia; CKD stage 2, CKD at IRIS stage 2. Statistical differences within each variable were analyzed using the Kruskal-Wallis test, with post hoc pairwise comparisons conducted using the Mann-Whitney U test. Categorical data were analyzed by Chi-square test. Significant differences (P < 0.05) between groups for each variable are indicated by same letters (a, b, c, d) within the same row. Groups sharing the same letter are significantly different. The results are presented as medians with interquartile ranges

| Parameters | Healthy  (n = 15) | MMVD B1  (n = 10) | MMVD C WOAZ  (n = 15) | MMVD C WAZ  (n = 13) | CKD stage 2  (n = 11) | P-value |
| --- | --- | --- | --- | --- | --- | --- |
| RBC sediment (cell/HPF) | 1[0 -2] | 1[0 - 2] | 1 [0 - 2] | 1[1 - 2] | 1[1 - 2] | 0.998 |
| WBC sediment  (cell/HPF) | 1 [0 - 2] | 3 [1 - 4] | 2 [0 - 4] | 4 [0 - 5] | 4[1 - 6] | 0.970 |
| Crystal | None (11),  Struvite (2), Calcium oxalate monohydrate (1), calcium oxalate dihydrate (1) | None (4),  Struvite (2), Calcium oxalate monohydrate (3), calcium oxalate dihydrate (1) | None (10), Struvite (3), Calcium oxalate monohydrate (2), | None (7),  Struvite (3), Calcium oxalate monohydrate (2), calcium oxalate dihydrate (1) | None (2),  Struvite (6), Calcium oxalate monohydrate (2), calcium oxalate dihydrate (1) | 0.62107 |
| Urine creatinine  (mg/dL) | 102 ±30.1 | 109.8 ±21.8 | 104.6 ±22.7 | 1.06.4 ±24.5 | 105.4 ±17.5 | 0.961 |
| Urine protein (mg/dL) | 6.5 [3.5 - 8.7] | 5.2 [3.7 - 6.65] | 4.5 [2.3 - 8.3] | 7.5 [2.9 - 8.8] | 7.1 [1.8 - 8.8] | 0.862 |
| UPCR | 0.09 [0.07 - 0.11] | 0.10 [0.07 -0.12] | 0.15 [0.12 - 0.18]^a^ | 0.15 [0.14 - 0.17]^a^ | 0.15 [0.13 - 0.18]^a^ | <0.001 |

Supplementary table 4 (cont). Urinalysis indices of the enrolled dog

Abbreviations: Healthy, healthy control; MMVD B1, MMVD dogs at stage B1; MMVD C WOAZ, MMVD stage C without azotemia; MMVD C WAZ, MMVD stage C complicated by azotemia; CKD stage 2, CKD at IRIS stage 2. Statistical differences within each variable were analyzed using the Kruskal-Wallis test, with post hoc pairwise comparisons conducted using the Mann-Whitney U test. Categorical data were analyzed by Chi-square test. Significant differences (P < 0.05) between groups for each variable are indicated by same letters (a, b, c, d) within the same row. Groups sharing the same letter are significantly different. Non-normal data are reported as medians with interquartile ranges, while normally distributed data are presented as means ± SD.
