# Supplementary material for: Serum peptidomic profiling and peptide mass fingerprinting reveal signatures associated with peroxisomal and mitochondrial pathways in MMVD-associated cardiorenal syndrome in dogs
Source: PLoS One. 2026 May 15;21(5):e0348233. doi: 10.1371/journal.pone.0348233 (PMC13178898; doi:10.1371/journal.pone.0348233)
Supplement: Attachment — Submitted filename: Response to the reviewers - PONE-D-25-68574_PP_SP.docx [file pone.0348233.s011.docx]

**Response to the reviewers:**

Thank you for the thorough and helpful review of our paper. We appreciate the opportunity to improve our work for publication in PLoS ONE. We have addressed all the concerns raised during the review, especially regarding the statistical replicates, animal ethics descriptions, and the scope of our conclusions. Please see our point-by-point responses and the changes made in the revised manuscript below.

**Reviewer: 1**

**1.** For nLC-MS/MS analysis, please provide details related to related to sample preparation (if also up to 10 kDa fractions were used), sample pooling and No. of technical replicates. From the Supplementary data it could be seen samples were analysed in triplicate. Please add this, otherwise the quantification results would not be confident if only one sample per group was used for statistical analysis. Furthermore, please clarify how many unique peptides were used for protein identification (line 270).

**Response:** Completed. We have revised and added information as suggested by the reviewer.

Lines 236-237. We used the <10 kDa peptide fractions obtained by ultrafiltration of the serum samples.

Line 267-268. To ensure the reliability and confidence of our quantification, each pooled group was analyzed in technical triplicate.

Line 283-288. For protein identification, a stringent filtering process was applied. Only peptides with a minimum length of seven amino acids were considered, and a minimum of two unique peptides per protein was required for identification. A 1% false discovery rate (FDR) at the protein level was maintained using a decoy (reversed sequence) database search. To further ensure identification accuracy, a maximum of five modifications per peptide was permitted.

We have clarified the protein identification criteria in the revised manuscript (Lines 283-288). Specifically, we required a minimum of two unique peptides for each protein identification, alongside a 1% protein FDR. Furthermore, following your suggestion, we have now included the actual number of unique peptides (**peptide count**) identified for each candidate protein in **Table 2** to provide greater transparency and confidence in our findings.

**2.** In the Table 2 the authors presented significant proteins, however GO data for several proteins is missing. Could you please update the table using available databases?

**Response:** We have updated Table 2 with additional Gene Ontology (GO) data using human orthologs where canine data was limited, offering a more comprehensive biological context for our results. (Lines 421).

**3.** It would be interesting to comment if some distinctive peaks (in terms of m/z) obtained by MALDI-TOF MS (PMF) were detected and/or identified by nLC-MS/MS. It would provide more strength to PMS results and its further clinical application.

**Response:** We appreciate the reviewer’s suggestion to correlate the MALDI-TOF MS (PMF) peaks with the nLC-MS/MS identifications. In our study design, these two techniques were used as complementary steps in a discovery pipeline.

MALDI-TOF MS was utilized as a high-throughput screening tool to evaluate the global peptide "fingerprint" and to verify the homogeneity of individual samples within each clinical group. This ensured that the subsequent nLC-MS/MS analysis was performed on representative pooled samples. While the PMF peaks provide a rapid means of group classification based on mass distribution, our primary objective for the nLC-MS/MS analysis was to perform a deep-dive into the peptidomic landscape to identify specific biomarkers and their associated pathways (e.g., mitochondrial and peroxisomal failure).

Although we did not perform a direct one-to-one assignment of MALDI peaks to peptide sequences, the nLC-MS/MS results provide the necessary biological identity to the peptidomic alterations observed during the initial screening. We have added a comment in the Discussion section (Lines 851-861) to clarify the distinct roles of these two mass spectrometry platforms in our study.

**4.** Please provide full name for CKD.

**Response:** chronic kidney disease (CKD) was added in Lines 31

**5.** Table 3. – Please change the title: Protein-drug pathway enrichment analysis performed using the Kyoto Encyclopedia of Genes and Genomes (KEGG). Furthermore, in FC enrichment and Enrichment FDR, please have in mind the number of decimal places, they should be the same within the column. I hope the text within the column names will be formatted/resized during paper publishing (e.g. Genes number, etc.).

**Response:** We thank the reviewer for these specific formatting suggestions to improve the readability and professional presentation of our data. We have implemented the following changes to Table 3:

- Title Update: The title has been changed to "Protein-drug pathway enrichment analysis performed using the Kyoto Encyclopedia of Genes and Genomes (KEGG)" as requested.
- Numerical Consistency: We have standardized the number of decimal places for both the Enrichment FDR and Fold Enrichment columns to four decimal places across the entire table to ensure consistency.
- Scientific Notation: Very small FDR values previously in scientific notation have been rounded to 0.0000 to maintain the uniform decimal format within the column.
- Column Formatting: We have adjusted the column headers and row alignment to ensure the table is clear and organized for the final publication layout.

**6.** Supplementary table 2 - Please correct: b Statistically significant difference compared Statistically significant difference compared between groups (P < 0.05).

**Response:** We apologize for the typographical error in the footnote of Supplementary Table 2. We have corrected the redundant text as suggested. The footnote now reads: "b Statistically significant difference compared between groups (P < 0.05)."

**Reviewer: 2**

1. The authors conclude that proteomic profiling and peptide mass fingerprints (PMFs) offer significant potential as auxiliary tools for the early detection of kidney disease in cardiorenal syndrome (CRS). However, in the present study, these proteomic profiling and PMF approaches identified renal pathology only in the two groups where conventional markers such as creatinine were already elevated. They did not identify renal pathology in MMVD non-azotemic dogs, where early renal disease initiation is still possible according to the CRS concept. Therefore, data demonstrating the renal marker potential of these proteomic signatures in subclinical renal disease associated with CRS would strengthen this conclusion.

**Response:** We agree with the reviewer’s assessment regarding the scope of our conclusions. We have revised the Conclusion section (Lines 867-883) to more accurately reflect that the identified proteomic signatures are associated with advanced cardiovascular-renal dysfunction (Type 2 CRS) rather than early subclinical detection.

2. The authors also state that canine cardiorenal syndrome is characterized by distinct serum peptide and protein alterations reflecting systemic failure of cellular redox and energetic homeostasis. However, the study evaluates only 13 cases belonging to Type 2 CRS (chronic heart disease with kidney injury; MMVD with azotemia) and does not prove cardiac pathology in chronic kidney disease cases (Type 4 CRS). It may therefore be inappropriate to generalize this pattern to the broader CRS condition when only one of the five CRS subtypes was studied with a limited sample size. It may be more appropriate to limit the conclusion to cardiovascular-renal dysfunction originating from cardiovascular disease (CvRDh). If the authors have data from other CRS groups, including them would help substantiate this broader conclusion. Additionally, the study does not establish peptide profiles indicating peroxisomal and mitochondrial dysfunction related to myocardial remodeling in MMVD without azotemia (WOAZ) cases.

**Response:** We concur with the reviewer’s evaluation of the extent of our conclusions. The Conclusion section (Lines 867-883) has been revised accordingly.

3. The study further concludes that the identified molecular changes persist despite standard medical therapy, suggesting that current treatments may not fully mitigate underlying organellar metabolic failure. However, this conclusion lacks supporting comparative data from untreated dogs, where proteomic changes might differ. Moreover, as the authors themselves mention, certain treatment protocols may augment azotemia, renal pathology, renin release, and subsequent RAAS activation. This could potentially exaggerate cardiac and/or renal peptide fragments in treated groups compared with untreated animals. Therefore, establishing this conclusion would require inclusion of treatment-naïve cases for comparison.

**Response:** We appreciate the reviewer’s critical point regarding the influence of medical therapy on the observed proteomic profiles. We agree that a comparison with treatment-naïve dogs would provide a clearer baseline to distinguish between disease-driven and treatment-induced molecular changes. However, as this was a clinical study involving client-owned animals presented to a veterinary teaching hospital, withholding standard medical therapy (such as ACE inhibitors, diuretics, or inotropes) from symptomatic dogs (Stage C MMVD) would have posed a significant ethical violation and compromised animal welfare. Consequently, our study aimed to reflect the real molecular landscape of canine cardiovascular-renal dysfunction under standard-of-care management.

We have revised our discussion (Lines 836-851) to clarify that the identified signatures demonstrate an association between current therapeutic protocols and the maintenance of certain metabolic pathways. We acknowledge that, as a limitation, we cannot definitively rule out whether some peptide fragments are exacerbated by the treatments themselves (e.g., drug-induced RAAS activation). We have added this clarification to the Study Limitations section to ensure a balanced interpretation of the results.

4. As mentioned above, data regarding the proteomic profile in cardiovascular disease/dysfunction secondary to renal disease (Type 4 CRS) and proteomic profile and PMFs in untreated MMVD Stage C dogs are not available in the manuscript. Inclusion of these datasets would help reinforce the conclusions.

**Response:** We fully agree with the reviewer that including proteomic profiles for Type 4 CRS (cardiovascular dysfunction secondary to renal disease) and treatment-naïve MMVD Stage C dogs would significantly strengthen the study and provide a more comprehensive overview of the cardiorenal continuum.

However, we were unable to include these datasets due to the following reasons:

- Ethical Constraints (Untreated Group): This study involved client-owned dogs presented for clinical treatment. As MMVD Stage C is a life-threatening condition, it was ethically mandatory to initiate standard-of-care therapy (e.g., diuretics, ACE inhibitors, inotropes) immediately upon diagnosis. Withholding treatment to obtain "treatment-naïve" proteomic data was not possible under our animal welfare protocols.
- Study Scope (Type 4 CRS): Our research was specifically designed as a prospective study focused on the progression of cardiovascular-renal dysfunction originating from primary heart disease (Type 2 CRS). While Type 4 CRS is a critical component of the cardiorenal syndrome, it represents a distinct pathophysiological direction that was outside the initial scope of this discovery-based study.

To address this comment, we revised the Discussion (Limitations section) and Conclusion to clarify that our findings are limited to Type 2 CRS and treated populations. We also highlighted the need for future studies including additional CRS subtypes to validate the role of mitochondrial and peroxisomal biomarkers across the cardiorenal syndrome spectrum.

Furthermore, this study did not include dogs with primary renal disease causing secondary cardiac dysfunction (Type 4 CRS) or symptomatic MMVD dogs prior to medical therapy. The following statement has been added to the manuscript: “The absence of these datasets limits the generalizability of our findings across the entire cardiorenal syndrome spectrum, and future research incorporating treatment-naïve cases and varying CRS subtypes is warranted to validate these proteomic signatures” (Lines 849–851). In addition, to better reflect the scope of the study, the manuscript title has been revised to “Serum peptidomic profiling and peptide mass fingerprinting reveal signatures associated with peroxisomal and mitochondrial pathways in MMVD-associated cardiorenal syndrome in dogs.” The Introduction has also been revised to clarify the study focus on MMVD-associated Type 2 CRS (Lines 79–96).

5. MMVD Stage C constituted an important sampling group in the present study. However, the Materials and Methods section does not clearly describe the criteria used for selecting this group. Details regarding the diagnostic criteria for MMVD Stage C, duration of illness, and treatment status should be incorporated. In addition, the authors should substantiate the reported normal systolic function findings in MMVD dogs that are described as having heart failure.

**Response:** We thank the reviewer for this insightful comment and agree that a clearer description of the MMVD Stage C group and further clarification of the systolic function findings are important for the interpretation of the study. Accordingly, the manuscript has been revised as follows:

- Details regarding the diagnostic criteria for MMVD Stage C, duration of illness, and treatment status have now been incorporated into the Materials and Methods section (Lines 122-132)
- Additional clarification has been added in the Discussion section to explain the preserved systolic function findings observed in MMVD dogs despite clinical evidence of heart failure (Lines 587–601). In addition, the limitations of this index have been discussed in the manuscript (Lines 822–827).

6. The proteomic profiles and PMFs identified were associated with metabolic dysfunctions relevant to renal pathology (CKD) and cardiorenal pathology (MMVD with azotemia). However, no data are presented to demonstrate that these markers are associated with isolated cardiac pathology, such as in MMVD Stage B1 or MMVD without azotemia. It is therefore possible that these proteomic signatures are present primarily in advanced renal (could be primary or secondary as in CRS) disease states, rather than in isolated cardiac disease or early renal pathology.

**Response**: We appreciate the reviewer’s insightful observation regarding the connection of these markers with advanced renal pathology. We agree that, based on our current data, the identified proteomic signatures (such as PEX14, GLRX2, and CKMT2) are most prominently expressed in groups with established renal impairment (CKD and MMVD with azotemia). This suggests that these markers may indeed reflect a critical threshold of metabolic and organellar failure that occurs when cardiovascular disease progresses to affect renal function. While we did not find significant alterations in isolated cardiac cases (Stage B1 or MMVD without azotemia), we believe this is an important finding in itself, as it highlights that the systemic failure of cellular redox and energetic homeostasis, involving peroxisomal and mitochondrial pathways, is a hallmark of the advanced cardiorenal continuum rather than early-stage cardiac remodeling alone.

We have revised our discussion to clarify that these signatures reflect combined cardiorenal dysfunction and may serve as markers for monitoring the transition from isolated heart disease to systemic cardiorenal failure. We have also addressed the limitation that these markers may not be sensitive enough for detecting isolated cardiac pathology or subclinical renal injury in the early stages of MMVD (Lines 832-834).

7. Although blood pressure was mentioned in the Materials and Methods section as a significantly differential parameter, the study does not present the blood pressure data for any of the study groups.

**Response:** We thank the reviewer for this valuable comment. Blood pressure data for all experimental groups have now been incorporated into the Results section (Lines 366–368). The corresponding interpretation and discussion have also been added to the discussion section (Lines 627-636).

Thank you very much.

(Poonavit Pichatapaiboon, DVM. MS)
Faculty of Veterinary Science, Mahidol University
